# Arbuscular mycorrhizal fungi increased biomass, nutritional value, and cochineal resistance of *Opuntia ficus-indica* plants

**DOI:** 10.1186/s12870-024-05432-7

**Published:** 2024-07-25

**Authors:** Teame Gebrehiwot Kebede, Emiru Birhane, Kiros-Meles Ayimut, Yemane G. Egziabher

**Affiliations:** 1 Department of Land Resource Management and Environmental Protection, College of Dryland Agriculture and Natural Resource, Mekelle University, Mekelle, Ethiopia; 2 Department of Animal Science, College of Agriculture and Environmental Science, Adigrat University, Adigrat, Ethiopia; 3 Institute of Climate and Society, Mekelle University, Mekelle, Ethiopia; 4 Department of Dryland Crop and Horticultural Science, College of Dryland Agriculture and Natural Resource, Mekelle University, Mekelle, Ethiopia; 5 Faculty of Environmental Sciences and Natural Resource Management, Norwegian University of Life Sciences (NMBU), Ås, Norway

**Keywords:** AMF inoculation, Cochineal stress, Drought stress, Nutrient digestibility, Macro and micro-nutrients

## Abstract

**Background:**

*Opuntia ficus-indica* (L.) Miller is dominantly growing on degraded soils in arid and semi-arid areas. The plants might establish a strong association with arbuscular mycorrhizal fungi (AMF) to adapt to nutrient, drought, and herbivore insect stress. The objective of this study was to investigate the effects of AMF inoculations and variable soil water levels (SWA) on the biomass, nutrient concentration, nutritional composition, and nutrient digestibility of the spiny and spineless *O. ficus-indica* by inducing resistance to cochineal stress. One mother *Opuntia ficus-indica* cladode was planted in a single pot in each field with 24 kg mixed soil. AMF inoculums were cultured in sorghum plants in a greenhouse and were inoculated in the planted cladodes. The planted cladodes were arranged using a completely randomized design (CRD) with three factors: AMF (present and absent); *O. ficus-indica* type (spiny and spineless) and four water treatments with 0–25% of plant available soil water (SWA), 25–50% of SWA, 50–75% of SWA, and 75–100% of SWA.

**Results:**

Drought stress reduced the below and above-ground biomass, cladode nutrient content, nutritional composition, and in vitro dry matter digestibility (IVDMD) and in vitro organic matter digestibility (IVOMD). AMF colonization significantly increased biomass production with significant changes in the macro and micro-nutrient concentrations of *O. ficus-indica*. AMF inoculation significantly increased the IVDMD and IVOMD of both *O. ficus-indica* types by improving the biomass, organic matter (OM), crude protein (CP), and reduced fiber and ash contents. AMF-inoculated cladodes improved the nutrient concentrations of the cladodes. AMF caused an increase in biomass production, increased tolerance to cochineal stress, and improved nutrient concentration, nutritional composition, and nutrient digestibility performance of *O. ficus-indica* plants.

**Conclusions:**

AMF improved the performance of the *O. ficus-indica* plant to resist drought and cochineal stress and increased the biomass, nutrient concentration, nutritional composition, and nutrient digestibility. The potential of *O. ficus-indica* to adapt to cochineal stress is controlled by the macro and micro-nutrient concentration brought by the AMF association.

## Introduction

The crassulacean acid metabolism (CAM) plants occur in 300 genera belonging to over 16,000 species [1]. and grouped into three subfamilies, namely *Opuntioideae*, *Pereskioideae*, and *Cactoideae* [2, 3]. *Opuntioideae* that has 220–350 species [3] are the largest and differ from all other *Cactaceae* in having glochids and seeds. *Opuntia ficus-indica* (L.) Miller is an important economic *Opuntioideae* species with delicious fruits and stems used for food and feed [4]. *O. ficus-indica* cladodes are cost-effective feed for ruminant animals because it is fast growing, easy and inexpensive to grow, edible, and able to survive prolonged droughts [5].

Nutrient content, nutritional composition, and digestibility of *O. ficus-indica* vary with age, cultivar types, species, and fertilization [6, 7]. For instance, *O. ficus-indica* types with few, short, and thin spines are higher in nutrient contents than long and thick spiny types [6, 8]. However, spiny and spineless types of *O. ficus-indica* are not significantly varied in mineral content and nutritional composition [9]. *O. ficus-indica* grows in the wild with low fertilizer, no irrigation inputs, and soils with low nutrient contents [8].

Animals found in arid and semi-arid zones get water-hydration benefits from feeding on cladodes of cactus [10] and improve their production performances [6]. Cladodes of the plants are high in carbohydrates, dry matter (DM), starch, and calcium [9]; water, ash, and energy [5]. On the other side, the crude protein (CP), crude fiber (CF), phosphorus (P), and nitrogen (N) content of the plant are low [8, 9, 11]. *O. ficus-indica* has a low CF, CP, P, and N content and high water content, and these limitations can lead to metabolic disorders, low DM intake, nutrient digestibility, diarrhea, and weight loss in animals [12, 13].

Proper interventions on cactus growth can improve the nutrition of cactus for animal feed. [11] studied the effect of silage making by spineless cladodes of *Opuntia stricta* associated with forages such as Buffel grass (*Cenchrus ciliaris* L.), *Gliricidia* (*Gliricidia sepium*) and *Pornunça* (*Manihot* sp.) on improving CF, CP, and N content, DM intake, and DM digestibility of the silage. Cladodes silage-making with other forage species had improved intake, digestibility, and nutrient contents. Mixing *O. ficus indica* with forage legumes can improve the nutrient contents of the feed diets and the silage [14]. *O. ficus indica* diets mixed with corn maize plants can improve protein stress and improve animal performance [15]. Mixing *O. ficus indica* diets with tef (*Eragrostis tef*) straw improves nutrient concentration and nutrient composition [16]. Interventions employed to improve the nutrient content of *O. ficus indica* help to improve the nutrient concentration, nutrient composition, and nutrient digestibility of the diet from the species. However, these studies focused on the improvement of the nutrient content and digestibility of the *O. ficus indica* plant when mixed with other plant species. *O. ficus indica* silage-making with protein-rich plants also demands inflated costs in terms of energy, sugar, water, labor, and handling [12]. AMF technology can independently improve the biomass and availability of nutrient contents in plants [17, 18]. Improving the nutrient content of plants is important to maximize digestive microbial efficiency and stimulate dry matter digestibility [19].

AMF is a key factor in promoting the primary productivity of plants [20] and nutrient content of woody plants [18, 21]. The role of AMF inoculation on the nutrient composition and nutrient digestibility of spiny and spineless *O. ficus indica* by inducing cochineal infestation resistance was not studied. *O. ficus indica* growing in arid and semi-arid areas is challenged by cochineal insects [22, 23]. Cochineal sucks the nutrients and water content of *O. ficus-Indica* plants [24] and brings poor nutritional quality of the plant and animal starvation effects [25]. In arid and semi-arid areas, interactions between plants and AMF play a significant role in inducing plant resistance to insect stresses and in maintaining the nutrient content of plant communities under insect stress [26]. AMF cochineal interactions in *O. ficus-indica* plants varied with different environmental factors [23]. Water stress impact on insect performance is complex and difficult, as water stress may have a positive, negative, or neutral effect on the performance of various herbivore insects [27, 28].

In our previous study, we concluded that colonization of *O. ficus-indica* plants with AMF has a major contribution to improved growth, biomass, and gas exchanges of the species [21]. However, nutrient concentration and nutritional quality improvements of *O. ficus-indica* through AMF inoculation need further study. A study of the underground process through AMF could help in the nutritional improvement of the *O. ficus-indica* and its digestible quality in a way that fulfills the needs of the livestock. In this study, we focused on the role of AMF symbiosis in improving the nutrient contents, nutritional values, and digestion of spiny and spineless *O. ficus-indica* under various soil water conditions (SWA). We hypothesized that: (1) mycorrhizal *O. ficus-indica* shows higher below and above-ground biomass productions, nutritional content, and composition than without AMF; (2) the nutritional content and composition of *O. ficus-indica* cladodes increase with an increase in soil water available; (3) mycorrhizal *O. ficus-indica* shows higher nutritional content, composition, in vitro digestibility and resistance against cochineal stress than non-mycorrhizal cladodes.

## Materials and methods

*O. ficus-indica* plants were cultured in the greenhouse of the Mekelle agricultural research center in Mekelle City, Tigray, Ethiopia (13°29′N, 39°28′E; 2000 m a. s. l.) for nearly two years before cochineal inoculation (from 10-Sep- 2019 to 14-Mar- 2021) and one year after cochineal infestation (from 15-Mar- 2021 to 20-Mar- 2022). The greenhouse had mean day/night temperatures of 26 °C/22°C. The mean daily average relative humidity of the greenhouse was 51%.

### Growing of *O. ficus-indica* plants

We collected one-year-old *O. ficus-indica* mother cladodes from the Mekelle research center. The mother cladodes were spiny and spineless. The morphological traits of the cladodes were not significantly varied [21]. We dried the cladodes under a shaded area for four weeks. We planted one air-dried cladode in an upright position in one pot filled with 23.5 kg of autoclaved river pure sand and field soil excavated from the rhizosphere of the *O. ficus-indica* plantation. The clay, silt, and sand soil textures for the potted soils were 12.8%, 7.1%, and 80.1% respectively. The potted soil had pH 7.77, electrical conductivity 0.11 dSm^− 1^, organic carbon 1.04%, total nitrogen 0.7%, available potassium 0.5 mg/100 g soil, and available phosphorous content 1.103 mg/100 g soil.

### Rhizosphere soil sampling, AMF spore extraction, cultivation, and application

We excavated soil samples from the rhizosphere of *O. ficus-indica* plantation following [23] procedures. AMF spore density and AMF genera types were evaluated using the excavated soil samples following [29] procedures. Potting soil was sieved and autoclaved at 121 ^O^C for two hours before inoculation. We cultivated AM fungi in pot cultures with *Sorghum bicolor* as the host plant. The viability of the sorghum seeds was determined before planting. Twelve sorghum seeds were planted in each pot and grown for 60 days. Soil and root samples were collected from each pot culture for further determination of AMF root colonization and spore density. Spore density and AMF root colonization were determined using a compound microscope with 400x magnification. The average spore density and AM fungi root colonization of the *Sorghum bicolor* plants 60 days after planting was 198.8 spore 100 g^− 1^soil and 99.21% respectively and these values were used to determine the weight of AMF inoculum added to the experimental pot. The fractional AMF root colonization and spore density from the AMF-inoculated *Sorghum bicolor* plants were determined and then the fungal inoculum and microbial wash were added to the center of each pot planted with mother cladodes [21]. The number of spores added to the center of each pot were close to 400 spores (198.8 spores × 201 g 100 g^− 1^ soil). Fungal inoculums were composed of a mixture of soil, spores, and root fragments, and 201 g of inoculum added to the center of each pot of the mother cladode. To mimic the optimum rhizosphere ecosystem and increase the AMF performance, 300 mL microbial wash created through the extraneous extraction solution (without spores) from fungi inoculums was added to the center of each pot. Adding 400 numbers of spores and 300 mL microbial wash is recommended for improved performance of plants and positive protection of insects [30].

### Greenhouse experimental design and treatments

In this study, a three-factorial experiment with two levels of AMF (presence and absence), two *O. ficus-indica* type (spiny and spineless), and four levels of plant available soil water (SWA), 0–25% of SWA representing T1, 25–50% of SWA representing a T2, 50–75% of SWA representing a T3, and 75–100% of SWA representing a T4 [21]. We arranged the treatments in a greenhouse workbench in a completely randomized design with seven replications giving a total of 112 *O. ficus indica* plants. In this study, an additional 12 controlled experimental pots filled with the same amount of mixed soil as the mother cladode planted pots were prepared to determine the exact percent of SWA [21] and we calculated the amount of water added to the planted pots following [31].

### Collecting ovipositing females, culturing, and inoculating crawler

We detached ovipositing females from cochineal-infested *O. ficus-indica* plants using a brush and collected in plastic jars [32]. The small female and male cochineals, crawlers, and dusty waxes were separated from the female cochineal by sieving. We cultured the alienated ovipositing females in plastic jars and kept them in a shaded area for 72 h. We produced enough crawlers for the treatments and sixty crawlers inoculated to the two sides of the treated cladodes on March 15/2021 following [33].

### Mycorrhizal root colonization analysis

The presence or absence of arbuscules, vesicles, and hyphae were used to assess mycorrhizal root colonization following the [34] method. We preserved the root samples collected from the *O. ficus-indica* plants in plastic jars filled with 50% ethanol [23, 34]. We chopped the collected root samples into 1 cm and treated them with 10% KHO solution in a heat-resistant jar. The treated roots were autoclaved at 121 °C for 15 min [29]. The roots were bleached and cleaned after washing in 10% H_2_O_2_ for about 15 min and acidified with 2% HCl for about 1 h at room temperature. We stained the roots in a mix of 0.05% trypan blue (5:1:1 lactic acid: glycerol: distilled water ratio). We washed the stained roots and immersed them in 50% glycerol for 1–2 h, for further de-staining and preservation. Afterward, the magnified gridline intersect method was used to determine the fractional AMF root colonization [29, 34].

### Biomass production

We uprooted the *O. ficus-indica* plants from the pots carefully. We carefully removed the roots from the uprooted plants to determine the below-ground biomass. Total biomass, shoot, and root biomass were determined by harvesting the whole plant. We then calculated both fresh and dry root-to-shoot ratio yields per plant. We washed the roots cut from *O. ficus-indica* thoroughly using water, cleaned them with a clean cloth to remove soil and then weighed using a sensitive balance. We recorded individual fresh weights for root and cladodes per plant. The roots and cladodes of *O. ficus-indica* were oven-dried at 100 ℃ for 24 and 72 h, respectively, until constant dry weight was achieved [31] and dry matter (g/kg fresh cladode of plants) was measured [35].

$$\:\text{D}\text{r}\text{y}\:\text{m}\text{a}\text{t}\text{t}\text{e}\text{r}=\:\frac{\text{F}\text{r}\text{e}\text{s}\text{h}\:\text{w}\text{e}\text{i}\text{g}\text{h}\text{t}-\text{D}\text{r}\text{y}\:\:\text{w}\text{e}\text{i}\text{g}\text{h}\text{t}}{\text{F}\text{r}\text{e}\text{s}\text{h}\:\text{w}\text{e}\text{i}\text{g}\text{h}\text{t}}\:\times\:100$$%

### Nutritional composition analysis

Before analysis, the grounded samples passed through a one mm sieve. We weighed three samples per treatment and stored them in sealed plastic bottles for laboratory analysis. The ash contents of the samples were determined by taking two grams of milled sub-samples and burned at 550 °C for three hours and we calculated the organic matter content by subtracting the percentage of ash from 100 [35]. The total nitrogen (N) content of all samples was determined by the Kjeldahl procedure. We calculated the crude protein content by multiplying the 6.25 factors with the nitrogen content of the cladodes [35]. The acid detergent fiber (ADF), acid detergent lignin (ADL), and neutral detergent fiber (NDF) content of samples were determined following the procedures of [36] by using filter-bag (Ankom^®^ Technology, # F57).

### In vitro digestibility

In vitro true dry matter digestibility (IVTDMD) and in vitro true organic matter digestibility (IVTOMD) are laboratory tests used as plant quality indexes for animal feed by animal nutritionists [37]. The rumen fluid was collected from three fistulated male sheep fat-ramped type (*Borona*) breed animals. The animals were fed twice a day with a basal diet combination of grass hay, agro-industrial by-products (wheat bran and molasses), and *O.ficus-indica* (chopped cladodes) based on their daily requirements. These basal diets were fed for seven days. Water was provided adlibitum. The rumen fluid was collected from the trial animals with a thermostat holding hot water at 39 °C. The fluid was added to the thermostat, after removing the water. IVTDMD and IVTOMD of all samples were determined by ANKOM (2008) Technology -DAISYII incubator using 0.25 g dried samples of *O. ficus-indica* passed through a one mm sieve size for IVTDMD analysis. The method includes two consecutive digestion phases. Plant materials were incubated under anaerobic conditions with rumen microorganisms for 48 h at 39 °C followed by a 24-hour acid-pepsin digestion phase at 39 °C during the first digestion phase. Residual plant materials were collected and oven-dried (105 °C for 12 h) after 72-hour incubation.

### Plant nutrient analysis

After calculating the dry weight of the plants, we cut plant samples into pieces using a sharp knife. We washed the cladodes cut from the *O. ficus-indica* plants thoroughly with distilled water and cleaned them with clean clothes to remove dust, cochineal, and cochineal products. The cut plants were dried. We crushed the dry cladode samples with a mortal and packed the resulting powder for the analysis of nutritional concentration, nutritional composition, and in vitro true dry matter digestibility. Nutritional concentration parameters: phosphorus (P), nitrogen (N), potassium (K), and calcium (Ca) were determined using an ultraviolet-visible spectrophotometry analyzer at Mekelle University Geology Laboratory and total magnesium (Mg), iron (Fe), manganese (Mn), and zinc (Zn) using atomic absorption spectrometry (AAS) at Ezana Analytical laboratory PLC, Mekelle, Tigray, Ethiopia. A triplicate of approximately five grams of the *O. ficus-indica* powder was placed into a 250 mL conical flask: 5 mL of concentrated H_2_SO_4_ was added following the addition of 25 mL of concentrated HNO_3_ and 5 mL of concentrated HCl. We heated the mixtures at 200 ^O^C for 1 h in a fuming hood and then cooled to room temperature. Then, 20 mL of distilled water was added, and we filtered the mixture using filter paper to complete the digestion of organic matter. Lastly, we transferred the mixture to a 50 mL volumetric flask, filled it to mark, and let it settle for at least 15 h. We analyzed Mg, Fe, Mn, and Zn using the resultant supernatant using [38].

### Statistical analysis

We analyzed the data for the root and cladode fresh weight, root and cladode dry weight, root/cladode ratio, DM, CP, ADF, NDF, ADL, and ash IVDMD content using the analyses of variance using SPSS 2016 software. Variations of AMF in *O. ficus-indica* daughter cladodes traits were evaluated using a three-way analysis of variance (ANOVA). Fractional root colonization of AMF in plant roots was evaluated using two-way ANOVA. We performed Gabriel *post hoc* test for unequal sample size and least significant difference (LSD) for the main effect comparison after checking for the normality of the distribution of the data. We estimated variability using standard errors and we used LSD (*α* = 0.05) to compare means at *P* < 0.05.

## Results

### Arbuscular Mycorrhizal Fungi colonization

**Fig. 1:**
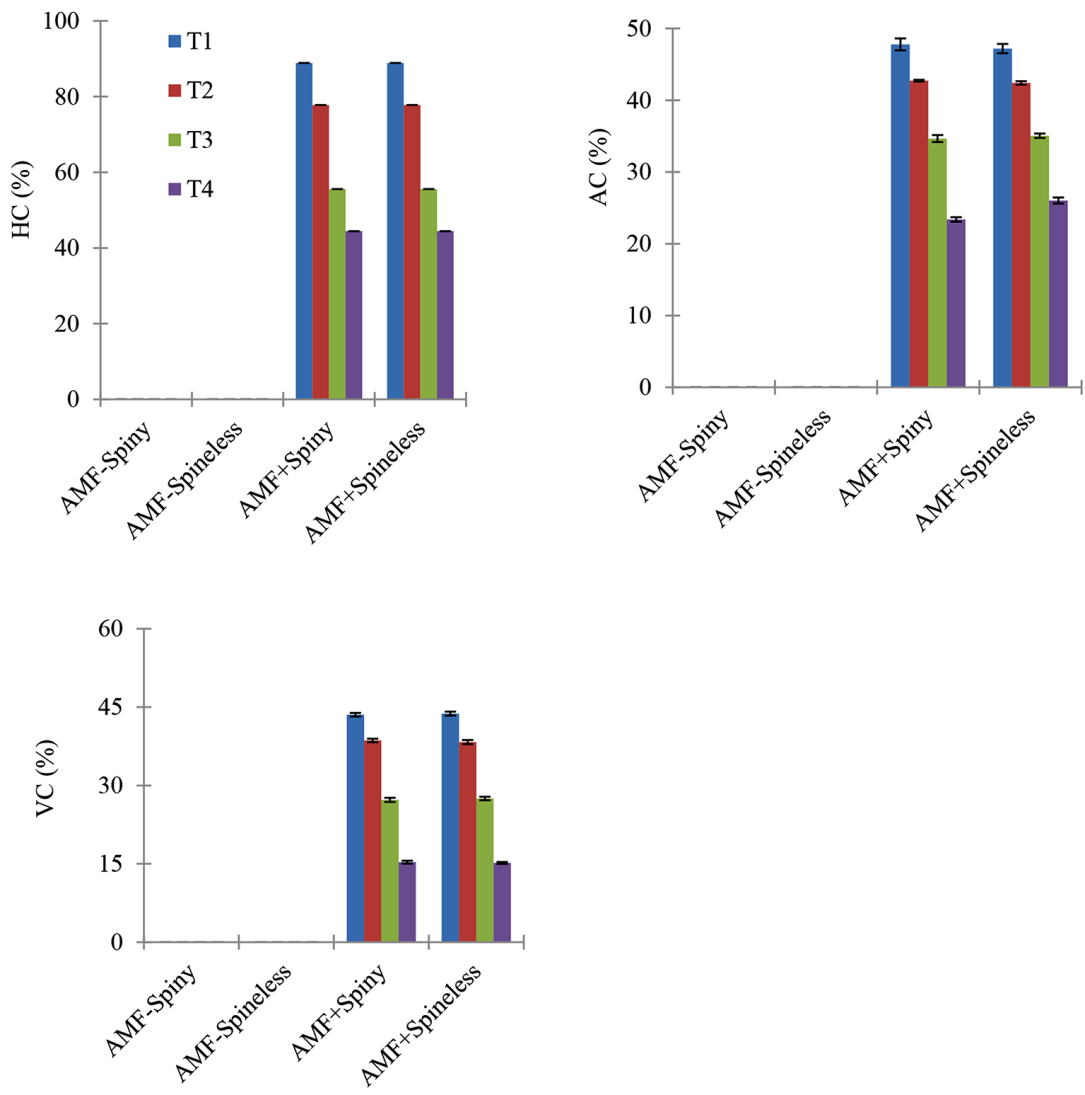
Effect of the interaction of *O. ficus-indica* type (spine and spineless), Arbuscular mycorrhizal fungi (AMF+, AMF-), and plant-soil water available (SWA) on fractional AMF root colonization HC = hyphal colonization, AC = arbuscular colonization, VC = vesicular colonization, and T1 = 0 to 25%, T2 = 25 to 50%, T3 = 50 to 75%, T4 = 75 to 100% of plant available soil water. Values indicate Mean ± SEM

Water treatment significantly affected the AMF root colonization (Table 1; Fig. 1). Root colonization significantly increased with increased drought stress. AMF root colonization was absent in non-AMF plants (Table 1). Presences of spines were not significant sources of variation.

### Biomass production

**Fig. 2:**
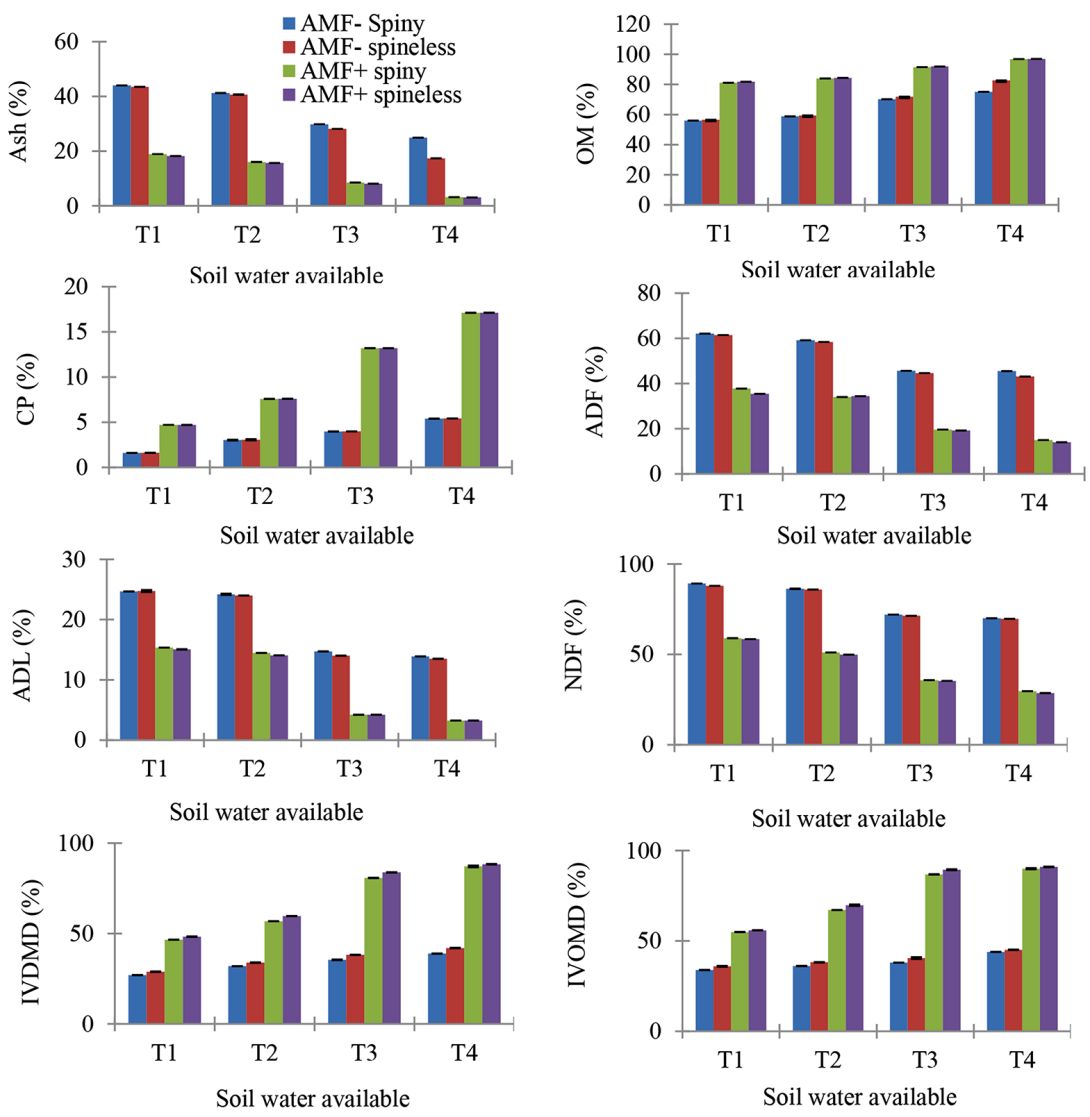
Effect of the interaction of *O. ficus-indic*a type (spine and spineless), Arbuscular mycorrhizal fungi (AMF+, AMF-), and plant-soil water available (SWA) on biomass production T1 = 0 to 25%, T2 = 25 to 50%, T3 = 50 to 75%, T4 = 75 to 100% of plant soil water available. Values indicate Mean ± SEM

AMF-inoculation and SWA significantly affected fresh mass, dry mass, and root/cladode ratio of *O. ficus-indica* plants (Table 1; Fig. 2). There was a significant interaction among *O. ficus-indica* type × AMF, *O. ficus-indica* type × SWA, AMF × SWA, and *O. ficus-indica* type × AMF × SWA in all biomass parameters. In this study, we observed that the below-ground interaction (*O. ficus-indica* type × AMF × SWA) affected the below and above-ground biomass of *O. ficus-indica* plants. Because the main inputs of the biomass of *O. ficus-indica* per pot is based on the effect of AMF accounts for the plant performance variance attributable to the difference between mycorrhizal and nonmycorrhizal *O. ficus-indica* plants.

**Table 1:** Effect of *O. ficus-indica* type, arbuscular mycorrhizal fungi (AMF), soil water available (SWA), and their interaction on the biomass and AMF colonization of *O. ficus-indica* plants

Factors		Biomass (g)							Root length colonization (%)		
		RFM	CFM	FR: FC	RDM	CDM	DR: CR	DM	HC	AC	VC
Types	Spiny	148.08 ± 16.89a	1190.03 ± 107.88a	0.110 ± 0.00a	68.45 ± 6.96a	324.04 ± 34.42a	0.235 ± 0.01a	75.00 ± 0.89a	35.35 ± 4.99a	18.91 ± 2.68a	15.87 ± 2.37a
	Spineless	158.89 ± 17.81a	1317.71 ± 114.34a	0.11 ± 0.003a	75.06 ± 7.58a	351.26 ± 37.24a	0.239 ± 0.01a	75.88 ± 1.01a	36.01 ± 5.04a	19.53 ± 2.71a	16.17 ± 2.4a
	*F*	0.051	0.132	0.733	0.279	0.615	0.506	0.720	0.101	0.156	0.079
	*P*	0.660	0.418	0.556	0.522	0.583	0.804	0.521	0.860	0.641	0.831
AMF	Presence	245.33 ± 15.47a	1923.8 ± 76.86a	0.03 ± 0a	102.31 ± 7.61a	540.71.8 ± 29.05a	0.17 ± 0.01b	78.12 ± 0.45a	69.44 ± 2.22a	37.4 ± 1.17a	31.19 ± 1.48a
	Absence	56.36 ± 4.2b	544.78 ± 29.8b	0.02 ± 0b	39.41 ± 2.94b	123.56 ± 7.91b	0.30 ± 0a	72.76 ± 1b	0b	0b	0b
	*F*	363.136	393.818	6.592	134.042	225.723	0.642	16.654	130.069	170.062	225.942
	*P*	< 0.001	< 0.001	< 0.001	< 0.001	< 0.001	< 0.001	< 0.001	< 0.001	< 0.001	< 0.001
SWA	T1	68.82 ± 21.09b	855.41 ± 144.85b	0.08 ± 0d	22.30 ± 7.47b	163.81 ± 45.47b	0.18 ± 0.013b	72.64 ± 0.83b	47.86 ± 7.08a	25.57 ± 3.81a	23.5 ± 3.27a
	T2	95.38 ± 20.7b	891.08 ± 142.14b	0.10 ± 03c	45.82 ± 7.33b	251.1 ± 44.62b	0.22 ± 0.013b	73.59 ± 1.1b	40.33 ± 6.95a	22.07 ± 3.74b	19.945 ± 3.21a
	T3	200.9 ± 20.32a	1545.1 ± 139.58a	0.12 ± 0b	97.37 ± 7.2a	437.37 ± 43.82a	0.26 ± 0.012a	75.14 ± 1.55b	33.33 ± 6.82b	17.43 ± 3.67bc	13.69 ± 3.15b
	T4	239.58 ± 20.33a	1680.1 ± 140.01a	0.13 ± 0a	116.96 ± 2.8a	483.69 ± 43.82a	0.28 ± 0.012a	80.39 ± 0.34a	22.22 ± 2.8b	12.35 ± 3.67c	7.63 ± 3.15c
	*F*	15.669	9.254	82.679	36.121	11.553	12.551	10.894	32.000	43.108	77.758
	*P*	< 0.001	< 0.001	< 0.001	< 0.001	< 0.001	< 0.001	< 0.001	< 0.001	< 0.001	< 0.001
Types × AMF	*F*	23.374	38.805	5.073	14.110	28.681	38.869	3.143	-	-	-
	*P*	< 0.001	< 0.001	0.008	< 0.001	< 0.001	< 0.001	0.053	-	-	-
Types × SWA	*F*	6.590	4.445	20.111	14.549	6.630	232.894	3.419	9.625	10.924	8.830
	*P*	< 0.001	0.001	< 0.001	< 0.001	< 0.001	< 0.001	0.011	< 0.001	< 0.001	< 0.001
AMF × SWA	*F*	409.938	128.158	483.417	577.165	260.174	19.049	73.238	-	-	-
	*P*	< 0.001	< 0.001	< 0.001	< 0.001	< 0.001	< 0.001	< 0.001	-	-	-
Types × AMF × SWA	*F*	28.887	24.090	32.365	45.545	27.898	4.598	2.660	39.472	36.145	24.841
	*P*	< 0.001	< 0.001	< 0.001	< 0.001	< 0.001	< 0.001	0.044	< 0.001	< 0.001	< 0.001

Means followed by the same letter in the same column are not significantly different at *P* < 0.05 level, mean ± SEM. Note: RFM: Root fresh mass, CFM: Cladode fresh mass, FR: CR: Fresh root to cladode ratio, RDM: Root dry mass, CDM: Cladodes dry mass, DR: CR: Dry root to cladode ratio, DM: Dry matter, HC: Hyphal Colonization, AC: Arbuscular Colonization, VC: Vesicular Colonization and T1: 0 to 25%, T2: 25 to 50%, T3: 50 to 75%, T4: 75 to 100% of plant soil water available

### Nutritional composition

**Fig. 3:**
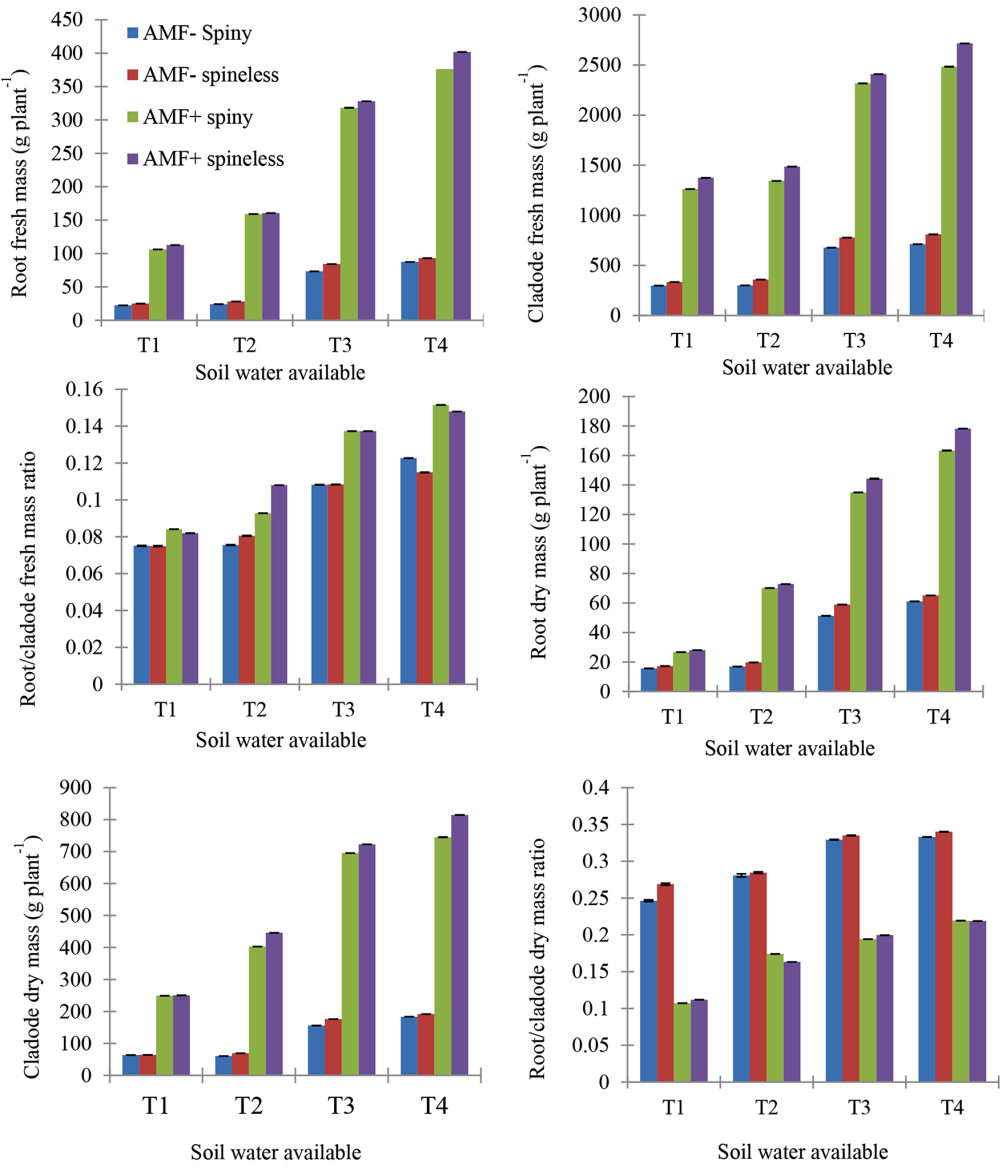
Effect of the interaction of *O. ficus-indica* type (spine and spineless), Arbuscular mycorrhizal fungi (AMF+, AMF-), and plant-soil water available (SWA) on nutritional composition and digestibility: OM = organic matter, CP = crude protein, ADF = acid detergent fiber, ADL = acid detergent lignin, NDF = neutral detergent fiber, IVDMD = in vitro dry matter digestibility, IVOMD = in vitro dry matter digestibility, and T1 = 0 to 25%, T2 = 25 to 50%, T3 = 50 to 75%, T4 = 75 to 100% of plant available soil water. Values indicate Mean ± SEM

The presence of spines in the cladodes did not have a significant effect on the nutritional compositions of the *O. ficus-indica* cladodes (Table 2). AMF and SWA had a significant effect on the nutritional compositions of the plants. Dry matter, organic matter, and crude protein content were significantly higher in AMF *O. ficus-indica* than in non-AMF *O. ficus-indica* plants. Ash content, ADF, ADL, and NDF were lower in AMF *O. ficus-indica* plants (Table 2; Fig. 3). An increase in SWA levels significantly increased the dry matter, organic matter, and crude protein content of the cladodes while decreased the Ash content, ADF, ADL, and NDF. *O. ficus-indica* type × AMF interactions significantly affected the nutritional composition in cladode ash content, OM, CP, ADF, ADL, and NDF (Table 2). The interactions between SWA and *O. ficus-indica* type significantly affected the DM, ash, OM, ADF, and ADL of daughter cladodes. *O. ficus-indica* type × AMF × SWA interaction significantly affected DM, ash, OM, CP, ADF, ADL, and NDF.

### In vitro digestibility

We assessed the effect of *O. ficus-indica* type, mycorrhiza, and plant-soil water available and their interaction on change in vitro dry matter (IVDMD) and in vitro organic matter digestibility (IVOMD) of *O. ficus-indica* cladodes after exposed to cochineal attack for twelve months. We found that AMF and SWA significantly affected the digestibility of cladodes (Table 2; Fig. 3). The non-mycorrhizal *O. ficus-indica* cladodes were lower in both IVDMD and IVOMD. There was significant interaction for *O. ficus-indica* type × AMF, AMF × SWA, and *O. ficus-indica* type × AMF × SWA for IVDMD and IVOMD.

**Table 2:** Effect of *O. ficus-indica type*, arbuscular mycorrhizal fungi (AMF), soil water available (SWA), and their interaction on the nutritional composition and digestibility of cladodes

Factors		Composition (%)						Digestibility (%)	
		Ash	OM	CP	ADF	ADL	NDF	IVDMD	IVOMD
Types	Spiny	23.33 ± 2.85a	76.67 ± 2.85a	4.13 ± 0.64a	39.84 ± 3.23a	14.35 ± 1.53a	61.61 ± 4.27a	50.55 ± 4.41a	56.31 ± 4.4a
	Spineless	21.84 ± 2.83a	78.16 ± 2.83a	4.13 ± 0.64a	38.83 ± 3.23a	14.11 ± 1.52a	60.87 ± 4.23a	52.88 ± 4.39a	58.17 ± 4.39a
	*F*	0.135	0.135	0.006	0.041	0.056	0.001	0.001	0.002
	*P*	0.855	0.855	0.848	0.801	0.875	0.896	0.992	0.998
AMF	Presence	11.46 ± 1.27b	6.48 ± 0.56a	88.54a	26.18 ± 1.97b	9.24 ± 1.15b	43.45 ± 2.45b	68.92 ± 3.44a	75.53 ± 3.02a
	Absence	33.71 ± 1.95a	1.78 ± 0.10b	66.29b	52.49 ± 1.64a	19.22 ± 1.1a	79.03 ± 0.03a	34.51 ± 1.01b	38.96 ± 0.77b
	*F*	11.535	11.535	114.153	8.261	5.486	12.246	97.982	126.590
	*P*	< 0.001	< 0.001	< 0.001	< 0.001	< 0.001	< 0.001	< 0.001	< 0.001
SWA	T1	31.16 ± 3.8a	68.84 ± 3.8b	6.26 ± 1.16a	49.19 ± 3.8a	19.96 ± 1.43a	73.63 ± 4.5a	37.67 ± 2.96c	45.17 ± 3.09c
	T2	28.41 ± 3.78a	71.59 ± 3.78b	4.94 ± 0.89ab	46.49 ± 3.7ab	19.19 ± 1.48a	68.27 ± 5.37ab	45.57 ± 3.84abc	52.71 ± 4.7abc
	T3	18.65 ± 3.12ab	81.35 ± 3.12ab	3.16 ± 0.47bc	32.26 ± 3.88b	9.29 ± 1.53b	53.6 ± 5.45ab	59.54 ± 6.87a	63.67 ± 7.34abc
	T4	12.13 ± 1.9b	87.87 ± 2.83a	2.15 ± 0.32bc	29.39 ± 4.5b	8.48 ± 1.58b	49.47 ± 6.14b	64.08 ± 7.14a	67.42 ± 6.91a
	*F*	6.653	6.653	5.465	6.242	16.844	4.577	4.925	3.101
	*P*	0.001	0.001	0.003	0.001	< 0.001	0.007	0.005	0.036
Types × AMF	*F*	13.805	18.805	1.520	13.498	7.228	14.521	13.643	15.884
	*P*	< 0.001	< 0.001	0.221	< 0.001	0.002	< 0.001	< 0.001	< 0.001
Types × SWA	*F*	2.783	0.029	1.096	2.557	0.042	2.273	1.463	2.273
	*P*	2.783	0.029	0.338	5.768	< 0.001	0.110	0.222	0.065
AMF × SWA	*F*	36.241	36.241	282.885	47.338	81.661	41.772	57.161	100.324
	*P*	< 0.001	< 0.001	< 0.001	< 0.001	< 0.001	< 0.001	< 0.001	< 0.001
Types × AMF × SWA	*F*	10.423	10.423	1.520	9.987	14.176	8.767	8.812	11.091
	*P*	< 0.001	< 0.001	0.203	< 0.001	< 0.001	< 0.001	< 0.001	< 0.001

Means followed by the same letter in the same column are not significantly different at *P* < 0.05 level, mean ± SEM. Note: OM: organic matter, CP: crude protein, ADF: acid detergent fiber, ADL: acid detergent lignin, NDF: neutral detergent fiber, IVDMD: in vitro dry matter digestibility, IVOMD: In vitro dry matter digestibility and T1: 0 to 25%, T2: 25 to 50%, T3: 50 to 75%, T4: 75 to 100% of plant soil water available

### Nutrient contents

**Fig. 4:**
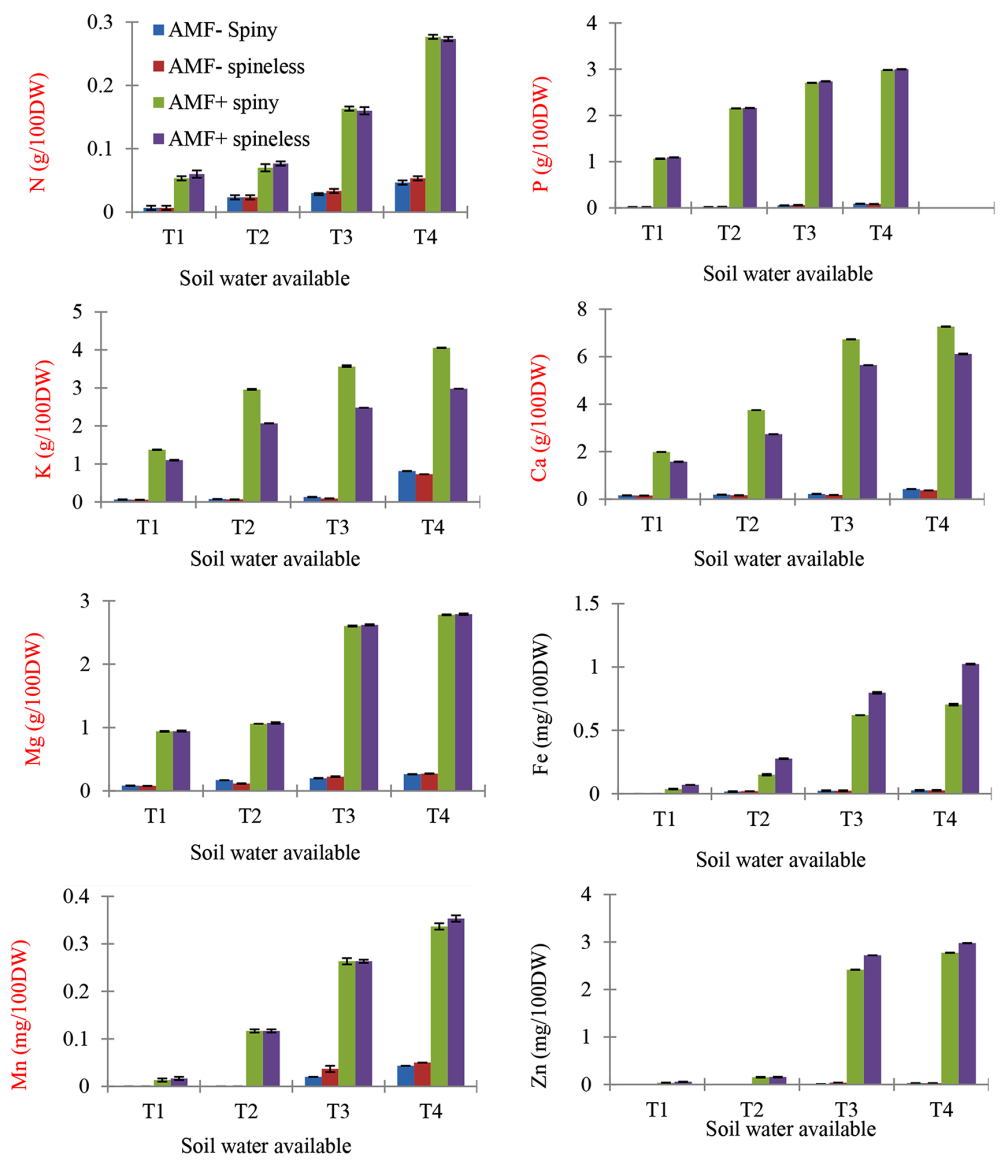
Effect of the interaction of *O. ficus-indica* type (spine and spineless), Arbuscular mycorrhizal fungi (AMF+, AMF-), and plant-soil water available (SWA) on nutrient composition: N = nitrogen, P = phosphorus, K = potassium, Ca = calcium, Mg = Magnesium, Fe = Iron, Mn = Manganese, Zn = Zinc, and T1 = 0 to 25%, T2 = 25 to 50%, T3 = 50 to 75%, T4 = 75 to 100% of plant available soil water. Values indicate Mean ± SEM

The P, N, K, Ca, Mg, Fe, Mn, and Zn nutrient concentrations in cladodes were significantly affected by AMF presence and SWA but not by *O. ficus-indica* types (Table 3; Fig. 4). The nutrient concentrations in cladodes were significantly higher for AMF-inoculated *O. ficus-indica* plants. Nutrient concentrations of the cladodes were increased with an increase in SWA levels. *O. ficus-indica* type was not a significant source of variation for nutrient concentrations. The P, Ca, Mg, Mn, and Zn concentrations were significantly affected by the interaction between *O. ficus-indica* type and SWA. All micro-nutrients (Fe, Mg, and Zn) were significantly affected by the interaction of *O. ficus-indica* type and SWA. AMF × SWA interaction significantly affected both macro and micronutrient concentration. *O. ficus-indica* type × AMF × SWA interaction significantly affected the nutrient concentrations except N and K.

**Table 3:** Effect of *O. ficus-indica* type, arbuscular mycorrhizal fungi (AMF), soil water available (SWA), and their interaction on the nutrient contents of cladodes

Factors		Macro- nutrients (g/100DW)					Micro-nutrients (mg/100DW)		
		N	P	K	Ca	Mg	Fe	Mn	Zn
Types	Spiny	0.84 ± 0.02a	1.14 ± 0.25a	1.63 ± 0.32a	2.59 ± 0.58a	1.01 ± 0.21a	0.2 ± 0.06a	0.1 ± 0.02a	0.68 ± 0.23a
	Spineless	0.09 ± 0.02a	1.15 ± 0.25a	1.2 ± 0.23a	2.12 ± 0.49a	1.02 ± 0.22a	0.28 ± 0.08a	0.1 ± 0.03a	0.75 ± 0.25a
	*F*	0.006	0.018	0.061	1.647	0.002	1.527	0.000	0.281
	*P*	0.848	0.932	0.744	0.535	0.993	0.424	0.883	0.840
AMF	Presence	0.14 ± 0.02a	2.24 ± 0.15a	2.57 ± 0.2a	4.47 ± 0.44a	1.85 ± 0.18a	0.46 ± 0.08a	0.18 ± 0.3a	1.41 ± 0.27a
	Absence	0.03 ± 0b	0.04 ± 0.01b	0.25 ± 0.06b	0.23 ± 0.02b	0.18 ± 0.01b	0.02 ± 0b	0.02 ± 0b	0.02 ± 0.4b
	*F*	114.153	373.042	132.525	152.230	207.983	153.328	102.302	183.751
	*P*	< 0.001	< 0.001	< 0.001	< 0.001	< 0.001	< 0.001	< 0.001	< 0.001
SWA	T1	0.03 ± 0.01b	0.55 ± 0.16b	0.65 ± 0.18b	0.97 ± 0.25b	0.51 ± 0.13b	0.03 ± 0.01b	0.01 ± 0c	0.02 ± 0.01b
	T2	0.05 ± 0.01b	1.09 ± 0.32ba	1.29 ± 0.38ab	1.71 ± 0.47b	0.6 ± 0.14b	0.12 ± 0.03ab	0.06 ± 0.01bc	0.08 ± 0.03b
	T3	0.1 ± 0.02ab	1.39 ± 0.4a	1.57 ± 0.45ab	3.19 ± 0.91a	1.41 ± 0.36a	0.37 ± 0.1ab	0.15 ± 0.07ab	1.3 ± 0.45a
	T4	0.16 ± 0.01a	1.54 ± 0.44a	2.14 ± 0.43a	3.54 ± 0.96a	1.53 ± 0.38a	0.44 ± 0.14a	0.2 ± 0.1a	1.45 ± 0.51a
	*F*	14.099	39.362	57.183	2.923	3.602	4.728	7.963	7.064
	*P*	< 0.001	< 0.001	< 0.001	0.044	0.021	0.006	< 0.001	0.001
Types × AMF	*F*	1.520	15.623	1.969	8.037	11.086	2.013	4.767	3.334
	*P*	0.221	< 0.001	0.164	0.001	< 0.001	0.145	0.013	0.045
Types × SWA	*F*	1.096	17.710	1.962	2.238	1.439	2.883	3.339	2.965
	*P*	0.338	< 0.001	0.146	0.068	0.230	0.025	0.013	0.022
AMF × SWA	*F*	282.885	390.091	67.495	224.930	103.658	18.316	224.126	304.750
	*P*	< 0.001	< 0.001	< 0.001	< 0.001	< 0.001	< 0.001	< 0.001	< 0.001
Types × AMF × SWA	*F*	1.520	7.863	0.826	9.230	7.752	6.531	9.469	7.909
	*P*	0.203	< 0.001	0.512	< 0.001	< 0.001	< 0.001	< 0.001	< 0.001

Means followed by the same letter in the same column are not significantly different at *P* < 0.05 level, mean ± SEM. Note: N: nitrogen, P: phosphorus, K: potassium, Ca: calcium, Mg: Magnesium, Fe: Iron, Mn: Manganese, Zn: Zink and T1: 0 to 25%, T2: 25 to 50%, T3: 50 to 75%, T4: 75 to 100% of plant soil water available

## Discussion

Our study supported the hypothesis that mycorrhizal *O. ficus-indica* shows higher below and above-ground biomass productions, nutritional content, and composition than without AMF. We observed a positive effect of mycorrhizal symbiosis on the below and above-ground biomass increment of *O. ficus-indica* under greenhouse conditions using spiny and spineless cladodes inoculated with AMF. Increased below and above-ground biomass in AMF plants compared to non-mycorrhizal ones reported for other species [18, 39]. The greater plant biomass measured from treatments inoculated with AMF could be due to enhanced nutrient uptake due to increased root surface area [40]. [21] reported that AMF inoculations enhance the biomass of spiny and spineless *O. ficus-indica* plants through improved growth, photosynthetic water use efficiency, and photosynthesis. [39] reported a positive mycorrhizal effect on the biomass of *Boswellia papyrifera* seedlings over control seedlings due to significantly improved growth, gas exchange, and P nutrition.

AMF inoculation caused a significant impact on biomass by the resistance of AMF to cochineal insects. Similarly, there are studies on enhanced biomass production of plants under insect stress due to AMF inoculation [23, 26, 30]. It is important to note that the above-ground biomass of the non-AMF-inoculated plants that was severely destroyed by the cochineal could have been altered by the sucking nutrients of the plant; therefore, we observed a strong difference in macro and micro-nutrients between the AMF-inoculated and controlled plants (Table 3; Fig. 4).

The biomass production significantly reduced with increased drought stress on both spiny and spineless plant types. Reduced biomass production of the plant in response to low levels of SWA has been well documented [21] and observed in CAM plants [41, 42]. This reduced biomass production performance is due to reductions in overall *O. ficus-indica* morpho-physiological performance [21]. However, AMF-inoculated plants were better adapted to drought stress than controlled plants. Similarly, [43] and [13] found that AMF-inoculated plants better induce tolerance to drought stress and improve biomass production than non-mycorrhizal plants.

For the first time, to our knowledge, we investigated the impact of AMF inoculation, SWA, and *O. ficus-indica* type on *O. ficus-indica* performance under nutrient-sucking cochineal insect stress. AMF inoculations effectively reduced the impact of cochineal on the *O. ficus-indica* biomass, nutrients, and national performance, while the SWA and types were not related to the cochineal impact. These responses are likely linked to changes in plant nutrients [32]. However, under cochineal-stressed conditions, the two- and three-way ANOVA interaction results showed that AMF, SWA, and types affected the plant performance measured parameters. In contrast to our observations, drought stress can only negatively affect the performance of western flower thrips, *Frankliniella occidentalis* (*Thysanoptera*: *Thripidae*), on tomatoes [44].

Our finding supported the hypothesis that the nutritional content and composition of *O. ficus-indica* cladodes increase with the increase in the amount of soil water available. Nutritional composition decreased with an increase in drought stress (Table 2). These results are consistent with the findings of [6], who investigated the effects of water stress on the chemical composition of spiny (*Opuntia amyclae*) and spineless (*Opuntia ficus indica f. inermis*) cactus cladodes. The result of this study showed that OM, CP, ADF, and lignin content were higher for water-stressed plants than for non-stressed plants. However, DM content was higher in non-stressed than water-stressed plants.

As hypothesized, we found higher IVDMD and IVOMD in the mycorrhizal *O. ficus-indica* cladode types than in non-mycorrhizal cladodes with the resistance of drought and cochineal stresses. These results can relate to the significant impact of AMF inoculation on the increment in weight, nutritive value, and nutritional composition (CP and OM) of the plant (Table 2). IVDMD and IVOMD in the *O. ficus-indica* cladode increased with an increase in biomass, nutritive value, and nutritional composition (CP and OM) and a reduction in fiber content, consistent with the findings of [16] of the plant. CAM plans have poor nutrient digestibility due to the low contents of crude protein (CP), crude fiber (CF), phosphorus (P), and nitrogen (N) [8, 9, 11]. However, AMF inoculation technology in this study improved the nutrient digestibility and improved the limited nutrient contents of *O. ficus-indica* plants.

## Conclusions

*O. ficus-indica* plants colonized by AMF improved the nutritional composition, nutrient digestibility, and mineral concentration by resisting cochineal and drought stress. AMF caused an increase in nutrient digestibility, which was related to the improvement of OM, CP, P, and N contents. A decrease in Ash, ADF, ADL, and NDF content improved IVOMD and IVDMD digestibility of the mycorrhizal *O. ficus-indica* plants. Cochineal stress brought low biomass production in the non-mycorrhizal plants. AMF-colonized *O. ficus-indica* plants inoculated with cochineal had better nutrient contents. AMF has interacted with decreasing levels of SWA. Thus, mycorrhizal *O. ficus-indica* plants with low levels of SWA increased their benefits for drought stress. AMF *O. ficus-indica* plants should be colonized by AMF to improve nutrient concentration, nutritional composition, and nutrient digestibility by resisting cochineal and drought stress.

## Data Availability

The relevant datasets supporting the results of this article are available with the authors and will be included within the article link at the respective time for the publication process.
